# Left Ventricular Hypertrabeculation and Ventricular Arrhythmias

**DOI:** 10.3390/jcm15093464

**Published:** 2026-05-01

**Authors:** Michele Alfieri, Samuele Principi, Alessandro Barbarossa, Federico Paolini, Lorenzo Torselletti, Francesca Coraducci, Sara Belleggia, Francesca Coretti, Paolo Compagnucci, Giulia Stronati, Michela Casella, Antonio Dello Russo, Federico Guerra

**Affiliations:** 1 Cardiology and Arrhythmology Clinic, Marche Polytechnic University, University Hospital “Umberto I–Lancisi–Salesi”, 60126 Ancona, Italy; m.alfieri@inrca.it (M.A.);; 2 Cardiology Unit, Istituto Nazionale di Ricovero e Cura per Anziani, Via della Montagnola 81, 60127 Ancona, Italy

**Keywords:** cardiomyopathy, left ventricular hypertrabeculation, left ventricular non-compaction, heart failure, sudden cardiac death, ventricular fibrillation, ventricular tachycardia

## Abstract

**Background/Objectives**: Excessive trabeculation of the left ventricle, previously known as left ventricular non-compaction (LVNC), is a rare phenotypic trait whose mechanisms and pathogenesis still remain conflictual. Its presentations may range from heart failure to embolism and, most importantly, ventricular arrhythmias (VAs). This study aims to find novel predictive factors for the occurrence of potentially fatal VAs in patients with left ventricular hypertrabeculation. **Methods:** All consecutive patients meeting the echocardiographic (Chin, Jenny or Stöllberger) and/or MRI criteria (Petersen) for hypertrabeculation were prospectively enrolled from October 2009 to December 2023. The primary outcome was a composite of sudden cardiac death, sustained ventricular tachycardias (sVTs), ventricular fibrillation (VF) or appropriate implantable cardioverter defibrillator (ICD) interventions. The secondary outcome was a composite of cardiovascular death and cardiovascular hospitalizations. **Results:** Overall, 64 patients (41 males, mean age 46 ± 19 years old) were enrolled and followed for a median time of 2.2 years. Six patients (9.4%) experienced a composite outcome at eight years, three with previous sVTs and three with previous non-sustained VTs (nsVTs). The strongest predictor of the primary endpoint was the anamnesis of nsVTs and sVTs before LVNC diagnosis. In addition, nsVTs and sVTs were significantly associated with the secondary outcome. **Conclusions:** Hypertrabeculation of the left ventricle is a complex and poorly understood condition whose status of cardiomyopathy is currently challenged. In our population, patients with a trabecular pattern experienced a high incidence of VAs, cardiovascular death and hospitalizations. VAs before LVNC diagnosis were predictive of the outcome independently from systolic function.

## 1. Introduction

Left ventricular hypertrabeculation, previously known as left ventricular non-compaction (LVNC) cardiomyopathy, represents a rare pathological entity characterized by the presence of endocardial prominent trabeculations and intertrabecular recesses communicating with the ventricular cavity [1,2,3].

While it was initially referred to as an “incomplete” maturation process from non-compacted to compacted myocardial walls, it is now accepted that compacted and trabeculated myocardium represent two different structures carrying distinct growth modalities; thus, the rationale to introduce the term “excessive trabeculation” in place of LVNC [4,5,6]. From a genetic standpoint, hypertrabeculation (OMIM #604169) is a highly heterogeneous condition. Pathogenic variants have been identified in sarcomeric genes, most notably MYH7 (beta-myosin heavy chain) and MYBPC3 (myosin-binding protein C), as well as in TTN (titin), SCN5A (cardiac sodium channel), RBM20 (RNA-binding motif protein 20), and TAZ (tafazzin), the latter being responsible for the X-linked Barth syndrome [2,7,8]. Of particular relevance, variants in the DES gene, encoding desmin, have been causally linked to LVNC. Desmin mutations associated with LVNC result in aberrant protein aggregation and severe disruption of filament assembly, as demonstrated by two specific pathogenic variants: the missense substitution p.A337P and a novel in-frame deletion within the 1A coiled-coil rod segment [9,10]. Furthermore, pathogenic LMNA variants, typically associated with arrhythmogenic dilated cardiomyopathy, can also present with a hypertrabeculated phenotype, reinforcing the concept that most LVNC-associated mutations are non-specific and shared across cardiomyopathy subtypes [2,8].

Some patients may present at an early age with neuromuscular, genetic (e.g., Barth syndrome [11]) or other congenital heart defects. In contrast, others may be referred later in the clinical course of the disease for heart failure, arrhythmias or even embolic events [12,13,14,15,16].

Despite the wide variability of the reported data, ventricular arrhythmias (VAs) and sudden cardiac death (SCD) seem to represent one of the leading causes of mortality, with an estimated incidence ranging up to 47% [17]. No specific recommendations are currently available to predict and prevent such unfortunate fatalities and, besides some indications [18], the decision to implant an implantable cardioverter defibrillator (ICD) still represents an open issue [19,20].

Among the most accredited mechanisms for VA development and perpetuation, the current literature depicts the presence of abnormal intercellular coupling, along with decreased coronary perfusion, which may represent the perfect substrate for focal and re-entrant ventricular activities [14,15,21,22,23]; however, this theory does not explain the high percentage of arrhythmias originating from non-trabeculated segments such as the right ventricular outflow tract [24,25].

The main aim of this paper is to identify the baseline predictor of sudden cardiac death (SCD) and sustained VTs in an LVNC population.

## 2. Materials and Methods

**Figure 1:**
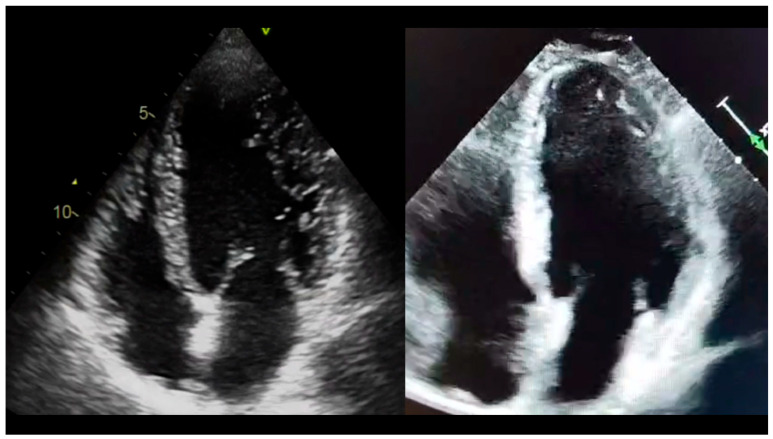
Four-chamber view of a patient with overt expression of cardiac hypertrabeculation (**left panel**) compared to a patient with a normal myocardial wall (**right panel**).

We prospectively and consecutively enrolled all patients referred to our Cardiology and Arrhythmology Clinic meeting the echocardiographic (Chin [26], Jenny [27] or Stöllberger [28]) or MRI criteria (Petersen [29]) for excessive trabeculation from June 2022 to November 2023. For patients with previous diagnosis of LVNC, permission was asked to retrospectively include all available data up from the date of first diagnosis, which fared back to October 2009. Figure 1 provides an example of one of our cases of LVNC compared to a normal myocardium, as seen by transthoracic echocardiography.

Exclusion criteria were the inability to sign informed consent for the study and comorbidities conditioning a life expectancy lower than one year. We also excluded patients with conditions potentially associated with a transient and benign hypertrabeculated pattern (e.g., aortic regurgitation, interventricular septal defects, athletes and peripartum cardiomyopathies [11,30]). All patients underwent a complete cardiological visit with an echocardiogram and ECG at baseline. Follow-up was conducted through a new visit every six months or through telephonic contact when the patient was not available. Genetic testing and MRI were conducted whenever possible. All patients were treated according to the proper guidelines and best medical practices. Due to an uneven rate of enrolment of our population (with a higher number of diagnoses made in recent years due to the creation of a specific clinic dedicated to rare diseases within our unit), follow-up was censored at eight years.

The present report complies with the STROBE initiative for observational studies (Table S1).

### 2.1. Endpoints

The primary outcome was a composite of sudden cardiac death (SCD) and sustained VTs (sVTs), defined as ventricular tachycardia lasting more than 30 s, ventricular fibrillation (VF), or appropriate ICD interventions.

The secondary outcome was a composite of cardiovascular death (including both sudden and non-sudden cardiac death) and cardiovascular hospitalizations. Cardiovascular hospitalizations were defined as any hospitalization longer than 12 h for one or more of the following reasons: acute coronary syndrome, heart failure, atrial or ventricular arrhythmias, valvular heart disease, infective endocarditis, myocarditis, pericarditis, aortic disease, pulmonary embolism, stroke/transient ischemic attack, syncope, cardiovascular-related elective and urgent procedures and complications of such procedures.

### 2.2. Data Collection

Data on patients’ demographics, risk factors, medical history, and treatment were collected prospectively by trained site staff. A comprehensive in-hospital visit, including a 12-lead ECG, blood test and an echocardiogram, was conducted at enrolment and every 6 months. Echocardiographic exams were performed by two trained cardiologists using Epiq7 systems (Philips Medical Systems, Amsterdam, The Netherlands), acquired with the patient in a supine position and left lateral decubitus at the end of a normal breath, aimed to minimize depth and optimize the frame rate (40–80 fps). LVEF was computed using the Simpson biplane method. Two authors conducted the offline analysis through the EchoPACS system (Philips, Amsterdam, The Netherlands), reviewing all echo exams and collecting measurements in a blinded manner to reduce intra-operator variability. Device interrogation was performed regularly at intervals lasting no longer than 6 months and personalized based on every patient’s specific needs; all patients carrying an ICD were also followed up through remote monitoring.

Magnetic resonance imaging (MRI) scans were performed in the same institution using a 1.5 T MR scanner (Achieva, Philips Medical Systems, Amsterdam, The Netherlands). The protocol included various imaging sequences, such as cine steady-state free-precession breath-hold sequences, T1-weighted breath-hold black-blood, and T2-weighted breath-hold black-blood short-tau inversion recovery sequences. Additionally, a phase-sensitive inversion recovery gradient-echo contrast-enhanced T1 sequence was acquired ten minutes after gadolinium injection, with the inversion time set to null normal myocardial signal intensity. Two expert physicians assessed the location and pattern of late gadolinium enhancement (LGE). All measured values were normalized based on body surface area.

### 2.3. Statistical Analysis

The normality of all continuous variables was examined using the Shapiro–Wilk test due to the small sample size. Variables exhibiting a normal distribution were characterized by mean and standard deviation. For non-normally distributed variables, median and 1st–3rd interquartile range (IQR) were used instead. Categorical variables were described in terms of absolute and relative values.

To evaluate the time free from primary and secondary endpoints, Kaplan–Meier analysis was employed. Due to a very heterogeneous follow-up time, with many patients being diagnosed in the most recent years, censoring after eight years was employed. In order to reduce overfitting due to the low number of events, univariate Firth’s penalized logistic regression hazard models were utilized to explore the association between individual variables and the primary and secondary endpoints, comparing the effect of the covariates by the Wald test. Variables demonstrating a significance level of <0.1 in univariate analyses for each endpoint were included in a multivariable Firth’s penalized logistic regression model. Independent risk factors for each endpoint were then presented as odds ratios (ORs) and 95% confidence intervals (CIs). Internal validation was performed using Harrell’s bootstrap optimism-correction method (B = 1000 resamples); optimism was estimated as the mean difference and subtracted from the apparent C-statistic to yield the corrected estimate. Calibration was assessed with the Hosmer–Lemeshow test using five groups, appropriate for this sample size.

The following covariates were tested as potential risk factors in all models: age, gender, arterial hypertension, dyslipidaemia, smoking status, cardiovascular medical history, history of VAs (both sustained and non-sustained), history of supraventricular arrhythmias, heart failure (HF) diagnosis, NYHA class, ejection fraction at baseline, non-compaction/compaction (NC/C) ratio at baseline, and number of NC segments at baseline.

SPSS 25.0 for Windows (SPSS Inc., Chicago, IL, USA) and R 4.4 (R Foundation for Statistical Computing, Vienna, Austria) were used for statistical analysis. Values of *p* < 0.05 (two-tailed) were considered statistically significant.

## 3. Results

### 3.1. Study Population

Overall, 64 patients (41 males, mean age 46 ± 19 years old) were enrolled after meeting the inclusion criteria. Forty-seven patients (73.4%) underwent an MRI and 36 (56.3%) met the Petersen criteria; 33 patients (64.7%) were positive only for echocardiographic findings, while 18 patients (51.6%) met both the MRI and echocardiographic criteria. Baseline characteristics are reported in Table 1. Median follow-up time was 2.2 years (IQR 0.9–6.5 years). No patients were lost during follow-up.

**Figure 2:**
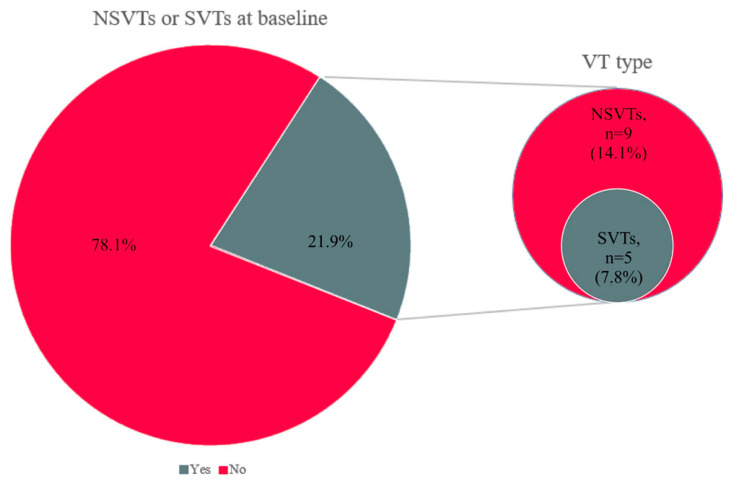
History of ventricular arrhythmias at enrolment.

Fourteen patients (21.9%) had a history of ventricular arrhythmias. Nine patients (14.1%) had a previous history of non-sustained VTs (nsVTs) detected during Holter monitoring or the stress test, while five (7.8%) had a positive anamnesis for sustained VTs (sVTs; Figure 2). All the latter were carriers of ICDs at enrolment, along with three other patients implanted for primary prevention (one patient in the nsVT group and the other two in the group without previous VTs).

**Figure 3:**
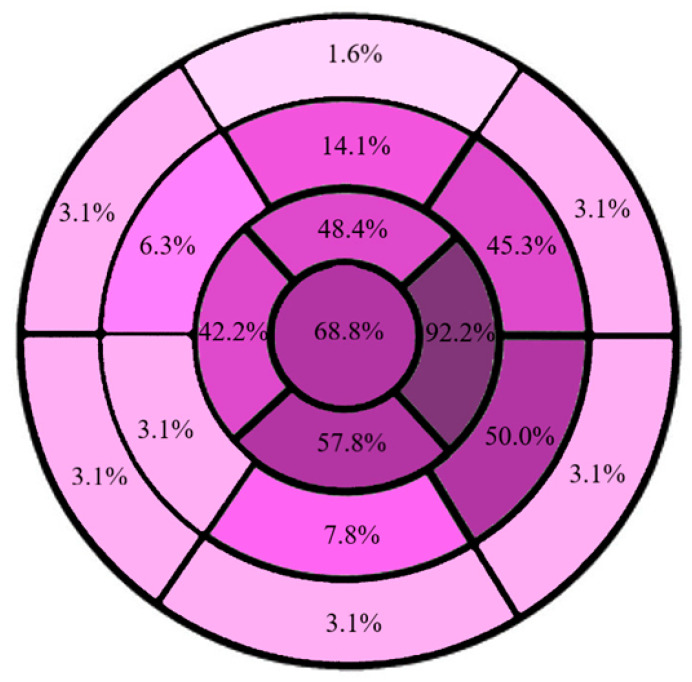
Prevalence of trabeculae localization according to left ventricular segments.

Patients with single or coupled ventricular ectopic beats not exhibiting nsVTs were included in the group with no arrhythmias at baseline. The localization of trabeculated segments in our population was identified by using the imaging technique (echo or MRI) with the highest diagnostic yield for each single patient. Late gadolinium enhancement (LGE) was present in 14 patients (21.9%), with the most common location in the antero-septal mid-ventricular segment (n = 7; 50.0%) with a prevalent mid-wall pattern; notably, LGE localization was not correlated with trabecular presence. In our sample, non-compaction follows a base-to-apex and septal-to-lateral gradient, with the most common presence in the apical lateral segment (>90%). No significant involvement of basal segments was observed (Figure 3).

### 3.2. Primary Outcome

**Figure 4:**
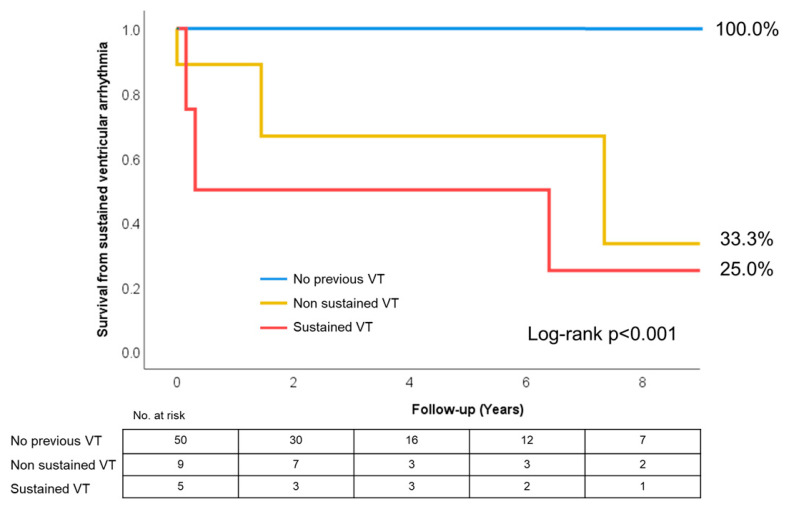
Kaplan–Meier curve for the primary outcome.

During the first eight years after diagnosis, six patients (9.4%) experienced the composite outcome: three in the sVT group and three in the nsVT group (Figure 4, log-rank *p* < 0.001). One patient (1.6%) in the nsVT group experienced VF, while the other two (3.6%) experienced tolerated sVT, and all three were implanted with an ICD (with one of those later receiving an appropriate ICD shock for VT recurrence). In the sVT group, three out of five patients experienced appropriate ICD interventions and two of them had multiple events during follow-up. In total, 53.8% (7 out of 13) of all appropriate interventions were detected with an LVEF of 50% and above. Remarkably, neither LVEF nor the presence of LGE or the NC/C ratio at baseline predicted the composite outcome.

Multivariate analysis (Table 2) shows that patients with anamnesis of VTs and nsVTs were, respectively, 33 and 13 times more likely to develop the composite outcome compared to patients with VEBs or without anamnesis of VTs. Of the 14 patients with either a history of sVT or an episode of sVT during follow-up, six (40%) underwent electrophysiological study and three (20%) were positive for VT induction. In all three cases, VTs from areas without hypertrabeculation could be detected.

The model demonstrated good apparent discrimination (C-statistic 0.897); after bootstrap optimism correction (optimism = 0.050), the corrected C-statistic was 0.847 (95% CI 0.713–0.978). The Hosmer–Lemeshow test showed no evidence of miscalibration (χ^2^ = 1.65, *p* = 0.648).

### 3.3. Secondary Outcome

During follow-up, 22 patients (34.4%) were hospitalized for cardiovascular reasons; three patients died, but there were no deaths due to known cardiovascular causes.

**Figure 5:**
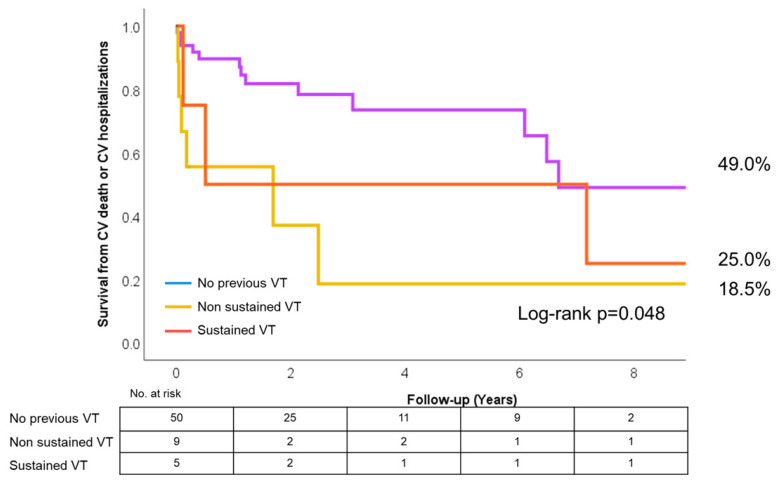
Kaplan–Meier curve for the secondary outcome.

Overall, survival from cardiovascular death or cardiovascular hospitalizations was 49.0% for patients without previous VT, 18.5% for patients with previous nsVT and 25.0% for patients with previous VT (Figure 5, log-rank *p* = 0.048).

## 4. Discussion

Our study gives a contemporary view of the clinical course and arrhythmic risk of patients with excessive trabeculation referred to a high-volume and high-specialty centre. The main results of the present study are as follows: (1) a history of VT, even non-sustained, is associated with an increased risk of both fatal arrhythmias, cardiovascular death and cardiovascular hospitalizations; (2) arrhythmic risk does not seem associated with systolic function, thus implying the need for a deeper evaluation in this subset of patients.

Left ventricular trabeculae are more present in apical and mid-lateral segments both by echocardiographic and cardiac magnetic resonance imaging. This pattern is similar to what has already been described in the literature [1,5,14,31] and strengthens the applicability of Stöllberger criteria in clinical practice. Nonetheless, as already postulated in a recent meta-analysis [8], hypertrabeculation does not seem associated with areas of increased electrical vulnerability or VT triggering, but, according to CMR studies, it correlates with a reduction in global deformation indices [32] and with a systolic disfunction directly proportional to the overall trabecular mass [33].

Arrhythmic events seem to represent one of the leading complications of such disease and our population reflects this pattern by showing a high percentage (11.8%) of patients experiencing the composite outcome.

Survival analysis, as represented in Figure 4, portrays two opposite sides of this population, with a cluster of patients showing a high propensity for the development of VTs. As expected, patients with anamnesis of sustained VTs experienced a composite outcome much more commonly than the rest of the group, with a risk 34 times higher, but, interestingly, even patients with nsVTs had a significantly higher (13 times) risk of developing VAs.

In our opinion, this observation might have a profound clinical impact, and it could be a key element leading us to the possibility of predicting major arrhythmic events, pushing the indications for ICD implantation even in primary prevention patients with non-sustained arrhythmic episodes and normal systolic function.

Conversely, among patients with premature ventricular complexes (PVCs), there were no events and the risk of experiencing the composite outcome was comparable to that of patients without arrhythmias at baseline. This observation reflects the results of another recent study confirming comparable survival for patients with and without PVCs in the absence of structural defects [34]. A possible explanation for this finding would be the presence of a different substrate able to organize extrasystoles into more complex arrhythmic phenomena, but data are still conflictual on this matter [34,35].

According to the 2019 Heart Rhythm Society Consensus [18], ICD implantation in primary prevention should be considered in patients with a history of nsVTs and reduced ejection fraction. This recommendation is based mainly on a large pediatric registry [36] and on a smaller adult prospective study [37] from 2009 to 2011. Since then, HF pharmacological treatment has made enormous leaps forward and has consistently provided beneficial effects on positive remodelling and arrhythmic risk [38,39].

In our sample, unlike other studies [8], left ventricular ejection fraction was not predictive of the composite outcome. This might be related to the restricted number of patients enrolled (imputable to the rarity of the disease) or could reflect a unique physio-pathological background of this condition for a marked propensity for the development of major ventricular arrhythmias, a propensity which is relatively independent of systolic function [9,13].

Several pathophysiological mechanisms may account for the dissociation between LVEF and arrhythmic risk in this phenotype. First, coronary microvascular dysfunction has been demonstrated in patients with isolated hypertrabeculation by positron emission tomography, suggesting that impaired subendocardial perfusion reserve, even in the absence of obstructive coronary artery disease, may create a chronically ischaemic, electrophysiologically heterogeneous myocardial milieu conducive to triggered activity and re-entry [22]. Second, diffuse interstitial and replacement fibrosis, detectable by parametric T1 mapping even in segments without focal LGE, has been increasingly recognized as an arrhythmic substrate in non-ischemic cardiomyopathies [40,41]. In hypertrabeculation, the abnormal trabecular architecture itself may foster mechanical stress and reactive collagen deposition across the entire ventricular wall, not merely in compacted segments. Third, autonomic imbalance, specifically augmented adrenergic tone, represents a trigger for ventricular arrhythmias in cardiomyopathies and may operate independently of systolic performance, potentially amplifying arrhythmic susceptibility in patients with preserved LVEF [42]. The coexistence of these mechanisms, acting on a background of abnormal cell-to-cell coupling inherent to the trabecular myocardium [21], may explain why nsVTs emerged as the dominant risk marker in our cohort and LVEF did not.

In fact, 43.8% of our patients had a diagnosis of HF at enrolment and 26.6% were affected by HFrEF. A total of 7.8% of patients experienced a decrease in EF to a value equal to or below 40% during follow-up and 45.5% of the population with HFrEF improved their systolic function during the observation period. Moreover, in our multivariable analysis, a previous diagnosis of HF, baseline EF, and NYHA class were not significantly associated with an increased risk of arrhythmias, and neither LVEF nor the NC/C ratio were predictive of the composite outcome. In addition, ICD recipients experiencing appropriate interventions had a concomitant reduction in LVEF only in 53% of all cases. These observations are in contrast with a recent meta-analysis on the matter [43] but are in line with the results of recent clinical studies such as the DANISH trial [44], which reported a low incidence of arrhythmic events in patients with non-ischemic heart failure. Furthermore, new evidence shows that, even in ischemic heart disease, most SCDs occur in patients with mildly reduced or preserved LVEF [45]. Thus, HF should not be the only factor taken into account when considering ICD in the primary prevention of sudden cardiac death for these patients, consistent with what is already suggested for other conditions such as HCM and LMNA-associated cardiomyopathies [20]. From our perspective, these findings point out that LVEF might be, at least in non-ischemic cardiomyopathies, the epiphenomenon of a subtle intracellular mechanism, predisposing to VAs, which may or may not be associated with a reduction in systolic performance. If our speculations are true, we still do not have efficient tools to detect such phenomena, but, at least in this population, the occurrence of nsVTs might be the game-changer we need. Furthermore, further insights from the DANISH trial show that, in non-ischemic heart failure patients, the presence of nsVTs carries a high prognostic burden and significantly correlates with cardiovascular death.

Another consideration is that, in our patients, LGE presence was not associated with the composite outcome. This contrasts with previous studies describing LGE as one of the main predictors of adverse events [46,47]. This lack of association might be imputable to the low number of patients and events, but, taking into account that MRI data could not be collected for all our patients, no clear conclusion can be made on this matter.

Alternatives to ICDs should be considered even in patients with hypertrabeculation. For example, Sohns et al. described ten patients undergoing catheter ablation [25] reporting acute procedural success in 90% of cases and a median time free from arrhythmia of 9.5 months. Of note, in this and other studies [24,48,49], coherently with our observations, the segments involved in VT origin were not always those usually presenting a trabecular pattern, according to our data (Figure 3). In addition, segments affected by hypertrabeculation were not reflective of the presence of LGE and localization. Therefore, it is feasible to hypothesize that LVNC represents the phenotype of a diffuse molecular substrate whose final expression is the presence of hypertrabeculation.

Our findings must be interpreted in the context of the conceptual shift introduced by the 2023 ESC guidelines [50], which deliberately refrain from classifying hypertrabeculation as a cardiomyopathy per se, instead repositioning it as a phenotypic trait that may occur in isolation, in the context of a defined cardiomyopathy, or as a benign anatomical variant. This reclassification carries direct implications for risk stratification and ICD decision-making. Under the previous paradigm, in which LVNC was treated as a discrete cardiomyopathy with intrinsically elevated arrhythmic risk, ICD implantation thresholds were frequently derived by analogy with dilated cardiomyopathy criteria, therefore principally relying on LVEF. The most recent guidelines effectively transfer the burden of proof to the clinician: the indication for an ICD must now rest on the individual arrhythmic risk profile rather than on the diagnosis of a specific disease label. Our data align with this logic. The fact that nsVTs predicted adverse outcomes independently of LVEF suggests that the arrhythmic phenotype, rather than systolic dysfunction, should drive ICD implantation in this population. In patients with hypertrabeculation who would not qualify for ICD implantation under conventional LVEF-based criteria for non-ischaemic cardiomyopathy, a history of nsVTs may therefore represent the critical risk discriminator that justifies primary prevention therapy. This interpretation is consistent with the risk-based, phenotype-agnostic approach advocated by the ESC 2023 guidelines and highlights the need for prospective registries capable of validating arrhythmic risk scores independent of ejection fraction in this population. Nonetheless, our data suggest that, regardless of its nosological status, hypertrabeculation is associated with an increased risk of fatal arrhythmias and, as such, should be followed up and stratified differently from the general population.

In conclusion, our analysis is one of the first attempts made to stratify the risk of VAs and SCD in patients with excessive trabeculation, an issue still largely unquestioned in this population; among others, nsVTs been have shown in this setting to be a strong indicator of such events, providing a useful tool to predict and, most importantly, prevent potentially fatal outcomes.

### Study Limitations

The main limitation of this study is the low number of patients, which resulted in a low incidence of the observed outcomes. The latter is particularly problematic, as having only six events vastly increases the risk of overfitting and imprecision of the logistic regression models, warranting caution in the interpretation of the ORs. Furthermore, the high number of patients enrolled in recent years of observation made the mean follow-up time overall heterogeneous. Special attention should be given to the fact that eight patients had an ICD implanted; thus, in this subgroup, event capture could probably be higher, especially regarding nsVT. In addition, not all patients went through a cardiac MRI due to various reasons (e.g., being already implanted at the time of enrolment) and we had no access to the genetic data; thus, the role of LGE and genetic applications are far from being clear in this study.

## 5. Conclusions

In conclusion, our analysis is one of the first attempts made to stratify the risk of VAs and SCD in patients with excessive trabeculation, an issue still largely unquestioned in this population; among others, nsVTs been have shown in this setting to be a strong indicator of such events, providing a useful tool to predict and, most importantly, prevent potentially fatal outcomes. Despite the 2023 ESC guidelines on the management of cardiomyopathies [47] not granting LVNC the status of cardiomyopathy, describing hypertrabeculation more as a phenotypic trait, our data suggest that these patients still have an increased risk of fatal arrhythmias and, as such, should be followed up and stratified differently from the general population. Nonetheless, the problem of phenocopies is still a significant hindrance and a proper tool for risk stratification is yet to come.

## Figures and Tables

**Table 1:** Characteristics of the population at baseline.

Characteristics	Total (n = 64)
Age, years, mean ± SD	46.5 ± 18.5
Male gender, n (%)	41 (64.1)
NYHA class, n (%)	
I	46 (71.9)
II	13 (20.3)
III	3 (4.7)
IV	2 (3.1)
Baseline EF, %, mean ± SD	49.5 ± 13.6
Baseline creatinine, mg/dL, mean ± SD	0.9 ± 0.2
Baseline eGFR, mL/min/1.73 m^2^, mean ± SD	103 ± 31
Comorbidities and risk factors	
Ischemic heart disease, n (%)	1 (2.0)
Arterial hypertension, n (%)	14 (21.9)
Dyslipidaemia, n (%)	15 (23.4)
Diabetes mellitus, n (%)	3 (4.7)
COPD, n (%)	2 (3.9)
Ongoing smoking habit, n (%)	8 (12.5)
Peripheral artery disease	2 (3.1)
Thyroid disease, n (%)	3 (4.7)
Anamnesis	
Previous stroke/TIA, n (%)	2 (3.9)
Previous VAs, n (%)	14 (21.9)
sVT, n (%)	5 (7.8)
nsVT, n (%)	9 (14.1)
SCD, n (%)	0 (0)
Previous SVAs, n (%)	11 (17.2)
Therapy at first assessment	
Beta-blockers, n (%)	33 (51.6)
ACE-inhibitors, n (%)	12 (18.8)
ARBs, n (%)	8 (12.5)
MRAs, n (%)	15 (23.4)
SGLT-2 inhibitors n (%)	4 (6.3)
ARNI	10 (15.6)
24/26 mg	3 (4.7)
49/51 mg	6 (9.4)
97/103 mg	1 (1.6)

ACE: angiotensin-converting enzyme; ARBs: angiotensin-receptor blockers; ARNI: angiotensin receptor–neprilysin inhibitor; COPD: chronic obstructive pulmonary disease; EF: ejection fraction; MRAs: mineralocorticoid receptor antagonists; nsVT: non-sustained ventricular tachycardia; SCD: sudden cardiac death; SGLT-2: sodium–glucose cotransporter 2; SVAs: supra-ventricular arrhythmias; sVT: sustained ventricular tachycardia; TIA: transient ischemic attack; VAs: ventricular arrhythmias; VEBs: ventricular ectopic beats; VF: ventricular fibrillation.

**Table 2:** Firth’s penalized logistic regression model for the primary outcome.

Variable	OR	95% CI Lower Bound	95% CI Upper Bound	*p* Value
No previous VT	Reference			
Non-sustained VTs	13.00	1.78	95.11	0.012
Sustained VTs	33.56	4.45	98.84	0.002
Age at enrolment	0.99	0.95	1.04	0.996
Male gender	4.48	0.55	36.77	0.740
LVEF	1.00	0.94	1.05	0.660

OR: odds ratio; VT: ventricular tachycardia; LVEF: left ventricular ejection fraction.

## Data Availability

Data can be made freely available by the authors upon reasonable request to the corresponding author.

## Supplementary Materials

The following supporting information can be downloaded at: https://www.mdpi.com/article/10.3390/jcm15093464/s1, Table S1: STROBE statement—checklist of items that should be included in reports of observational studies.

## Abbreviations

The following abbreviations are used in this manuscript:

ECG	Electrocardiogram
EF	Ejection fraction
HF	Heart failure
ICD	Implantable cardioverter defibrillator
IQR	Interquartile range
LGE	Late gadolinium enhancement
LVNC	Left ventricular non-compaction
MRI	Magnetic resonance imaging
NC/C	Non compaction/compaction ratio
NYHA	New York Heart Association
PVC	Premature ventricular complex
VT	Ventricular tachycardia
VF	Ventricular fibrillation
